# Characterization of Adult α- and β-Globin Elevated by Hydrogen Peroxide in Cervical Cancer Cells That Play A Cytoprotective Role Against Oxidative Insults

**DOI:** 10.1371/journal.pone.0054342

**Published:** 2013-01-17

**Authors:** Xiaolei Li, Zhiqiang Wu, Yao Wang, Qian Mei, Xiaobing Fu, Weidong Han

**Affiliations:** 1 Department of Molecular Biology, Institute of Basic Medicine, School of Life Sciences, Chinese PLA General Hospital, Beijing, China; 2 Department of Immunology, Institute of Basic Medicine, School of Life Sciences, Chinese PLA General Hospital, Beijing, China; University of California-San Francisco, United States of America

## Abstract

**Objectives:**

Hemoglobin (Hgb) is the main oxygen and carbon dioxide carrier in cells of erythroid lineage and is responsible for oxygen delivery to the respiring tissues of the body. However, Hgb is also expressed in nonerythroid cells. In the present study, the expression of Hgb in human uterine cervix carcinoma cells and its role in cervical cancer were investigated.

**Methodology:**

The expression level of Hgb in cervical cancer tissues was assessed by quantitative reverse transcriptase-PCR (qRT-PCR). We applied multiple methods, such as RT-PCR, immunoblotting, and immunohistochemical analysis, to confirm Hgb expression in cervical cancer cells. The effects of ectopic expression of Hgb and Hgb mutants on oxidative stress and cell viability were investigated by cellular reactive oxygen species (ROS) analysis and lactate dehydrogenase (LDH) array, respectively. Both Annexin V staining assay by flow cytometry and caspase-3 activity assay were used, respectively, to evaluate cell apoptosis.

**Results:**

qRT-PCR analysis showed that Hgb-α- (HBA1) and Hgb-β-globin (HBB) gene expression was significantly higher in cervical carcinoma than in normal cervical tissues, whereas the expression of hematopoietic transcription factors and erythrocyte specific marker genes was not increased. Immunostaining experiments confirmed the expression of Hgb in cancer cells of the uterine cervix. Hgb mRNA and protein were also detected in the human cervical carcinoma cell lines SiHa and CaSki, and Hgb expression was up-regulated by hydrogen peroxide-induced oxidative stress. Importantly, ectopic expression of wild type HBA1/HBB or HBA1, rather than mutants HBA1^H88R^/HBB^H93R^ unable to bind hemo, suppressed oxidative stress and improved cell viability.

**Conclusions:**

The present findings show for the first time that Hgb is expressed in cervical carcinoma cells and may act as an antioxidant, attenuating oxidative stress-induced damage in cervical cancer cells. These data provide a significant impact not only in globin biology but also in understanding of cervical cancer pathogenesis associated with oxidative stress.

## Introduction

Hemoglobin (Hgb), the major heme protein of erythrocytes, facilitates the transport of oxygen and carbon dioxide in the blood. The Hgb molecule is an assembly of four globular protein subunits. HgbA, the most common type of Hgb in adult humans, is a tetramer consisting of two heme-containing α- and β-subunits held together by noncovalent interactions (α_2_β_2_) [1], [2]. Other vertebrate heme containing proteins with structural homology to globin chains include myoglobin, which is widely found in vertebrate hearts and striated and smooth muscles [3], cytoglobin, mostly described in connective tissues [4], and neuroglobin, broadly expressed in the brain [5].

The common function of Hgbs as a vertebrate main oxygen and carbon dioxide carrier is a relatively recent adaptation during evolution, required to ensure correct delivery of oxygen to all cells of the body via means of the vascular network. In addition to this classical role, Hgbs can perform other cellular activities, including intracellular oxygen transport, oxygen sensing, NO scavenging, hydrogen peroxide scavenging, and iron metabolism regulation [6]. Recent studies reported the detection of Hgb chains in other cells than those of the erythroid series, including neurons [7], [8], [9], retinal cells [10], [11], alveolar epithelial cells [12], [13], [14], endometrium [15], rat kidney mesangial cells [16], hepatocytes [17] and macrophages [18]. It was speculated that the function of Hgb in neurons could be oxygen storage [9]. In alveolar epithelial cells, hypoxia-induced Hgb expression may play a role in the oxygen sensing pathway [14]. Microarray analyses of MN9D dopaminergic cell lines stably transfected with Hgb-α-globin (HBA1) and Hgb-β-globin (HBB) chains revealed changes in the expression of genes involved in oxygen homeostasis and mitochondrial oxidative phosphorylation [7]. Moreover, Hgb overexpression was shown to reduce oxidative stress, suggesting that Hgb functions as an antioxidant [16], [17].

Oxidative stress is caused by an imbalance between the formation of active oxygen metabolites and the rate at which they are scavenged by enzymatic and non-enzymatic antioxidants, and it has been associated with the pathogenesis and complications of several diseases, including cancer [19]. The overproduction or inadequate removal of reactive oxygen species (ROS) such as hydrogen peroxide, hydroxyl radicals and superoxide anion is associated with carcinogenesis [20] and invasiveness [21]. Enzymatic and non-enzymatic antioxidant defense mechanisms regulate cellular redox balance and therefore the pathogenesis of many diseases. However, our understanding of the roles of oxidant and antioxidant systems in carcinogenesis and tumor development remains limited. Remarkably, a recent study showed that Hgb functions as an antioxidative peroxidase, attenuating hydrogen peroxide induced oxidative stress [22].

Advances in high-throughput microarray technology have enabled the comparison of gene expression profiles between primary tumors and normal tissues. Microarray-based gene expression analyses identified Hgb genes differentially expressed in solid tumors including breast cancer [23], ovarian serous carcinoma [24], and colorectal carcinoma [25]. A recent proteomic study showed that serum HBA1 and HBB levels were significantly elevated in ovarian cancer patients compared to normal healthy females [26]. Although the source of free Hgb in the serum was unknown, it was assumed to be the result of oxidative stress-induced hemolysis. The mechanisms underlying the regulation of Hgb and its functional consequences in solid tumors remain to be fully understood.

In the present study, we show that Hgb-α, adult chain 1 (Hba-a1), and Hgb-β, adult chain 1 (Hbb-b1) are expressed in carcinoma cells of several types of solid tumors. Hgb expression was detected in clinically resected specimens of human cervical tissue and was up-regulated in cervical cancer tissues compared with normal tissues. Furthermore, our results indicated that oxidative stress up-regulates Hgb expression. Importantly, Hgb overexpression reduced oxidative stress and improved the viability of cervical cancer cells. Collectively, our results suggest that Hgb may be a component of the endogenous antioxidant defense system, protecting cells against oxidative damage in patients with cervical cancer.

## Results

### Increased Hgb Gene Expression in Cervical Cancer Specimens

To investigate differentially expressed genes in cervical cancer, we analyzed a previously published microarray dataset including 33 cervical cancer and 24 normal samples. Compared to normal cervix epithelium, cervical cancer samples showed a significant elevation in the expression of HBA1 and HBB genes (Table 1). However, the expression of transcription factors for erythroid differentiation [27], including NFE2, KLF1/EKLF, TAL1/SCL and GATA1 did not increase. In addition, the expression of other hemoglobin genes (HBD, HBE1, HBG2, HBQ1 and HBZ) and erythrocyte specific marker genes, such as SPTB, ERAF and ALAS2 did not show a significant increase. These results suggest that elevated HBA1 and HBB expression in cervical cancer did not result from erythropoiesis, but from a different mechanism.

**Table 1:** Elevated HBA1 and HBB expression in cervical cancer.

Gene Name	Gene Symbol	Cervical Cancer		Normal Cervix Epithelium	Fold Change^a^		*P* Value					
**Hemoglobin family**												
Hemoglobin, alpha	HBA	3806.24		2149.13	1.77		0.037					
Hemoglobin, beta	HBB	3505.32		2364.09	1.48		0.043					
Hemoglobin, delta	HBD	54.85		63.46	0.86		0.523					
Hemoglobin, epsilon 1	HBE1	44.57		44.89	0.99		0.965					
Hemoglobin, gamma G	HBG2	220.53		192.29	1.15		0.691					
Hemoglobin, theta 1	HBQ1	3.75		5.84	0.64		0.006					
Hemoglobin, zeta	HBZ	1.98		3.43	0.58		0.002					
**Transcription factors for erythroid differentiation**												
Nuclear factor (erythroid-derived 2)	NFE2		18.09	17.52		1.03		0.946				
Kruppel-like factor 1 (erythroid)	KLF1/EKLF		37.76	46.48		0.81		0.176				
T-cell actue lymphocytic leukemia 1	TAL1/SCL		50.71	61.59		0.82		0.112				
GATA binding protein 1 (globin transcriptionfactor 1)	GATA1		35	44.92		0.78		0.257				
**Erythroid cell markers**												
Spectin, beta, erythrocytic	SPTB		65.29	58.93		1.11			0.525			
Erythroid-associated factor	ERAF		43.95	67.70		0.65			0.007			
Aminolevulinate, delta-, synthase 2	ALAS2		6.78	7.28		0.93			0.828			

a Fold-change is calculated based on comparison between cervical cancer and normal cervix epithelium.

**Figure 1:**
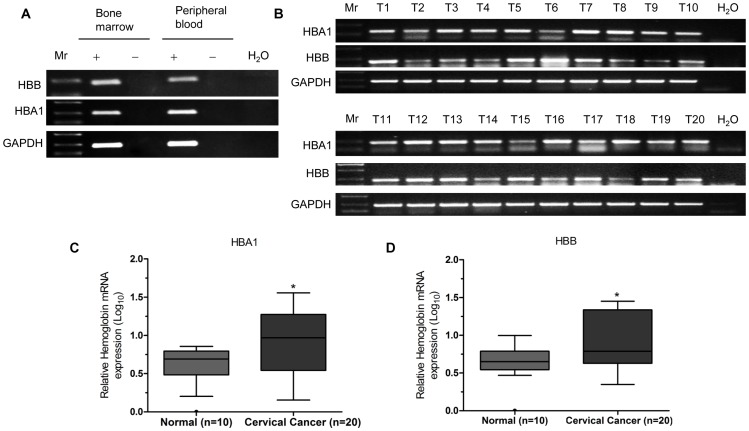
Elevated expression of HBA1 and HBB in human cervical cancer tissues. (**A**) To confirm the specificity of the HBA1 and HBB primers for qRT-PCR, expression of HBA1 and HBB was examined in human bone marrow and peripheral blood cells. Retrotranscriptase free (–) as a negative control, H_2_O as a system control. Mr, Marker. (**B**) cDNA from cervical cancer samples was prepared and analyzed for expression of HBA1 and HBB by RT-PCR. PCR products were separated on 2% agarose gels and visualized with ethidium bromide. GAPDH was used as a loading control. H_2_O as a system control, T, represents primary tumour samples. The expression levels of HBA1 and HBB were measured by qRT-PCR in 20 human cervical cancer specimens and 10 normal cervical tissue controls. Quantification of the indicated normalized HBA1 and HBB levels (log_10_) of the total unpaired normal cervix (n = 10) and cervical cancer (n = 20) samples are shown. The tukey boxes represent the upper and lower quartiles divided by the median and whiskers are the largest and smallest values, excluding outliers represented by circles. All differences were statistically significant at *P*<0.05 (**C and D**).

In order to validate the potential role of Hgb in human cervical carcinoma, the expression levels of HBA1 and HBB were investigated in 20 cervical cancer specimens and 10 normal cervix tissue samples by qRT-PCR analysis. The specific primers of HBA1 and HBB were designed for qRT-PCR according to the National Center for Biotechnology Information (NCBI) database. The specificity of the primers for HBA1 and HBB amplification was confirmed using RNA extracted from human bone marrow and peripheral blood as a positive control (Fig. 1A). Firstly, RT-PCR experiment was performed to determine the expression levels of HBA1 and HBB genes in 20 cervical cancer samples (Fig. 1B). Consistent with the microarray dataset, qRT-PCR analysis showed that the expression of HBA1 and HBB was significantly higher in cervical cancer tissues than in normal cervix tissues (Fig. 1C and D). However, the expression of transcription factors for erythropoiesis, including GATA-1, KLF1, and SCL/TAL1 [27], was not changed significantly in cervical cancer tissues compared to the controls (data not shown). The expression of erythrocyte specific marker genes, such as SPTB, ERAF, was not up-regulated in cervical cancer tissues, indicating that the increase in Hgb expression was not associated with erythropoiesis. These results suggest that Hgb may play a role in human cervical cancer development.

### Detection of the Hgb Protein in Cervical Carcinoma Tissues

**Figure 2:**
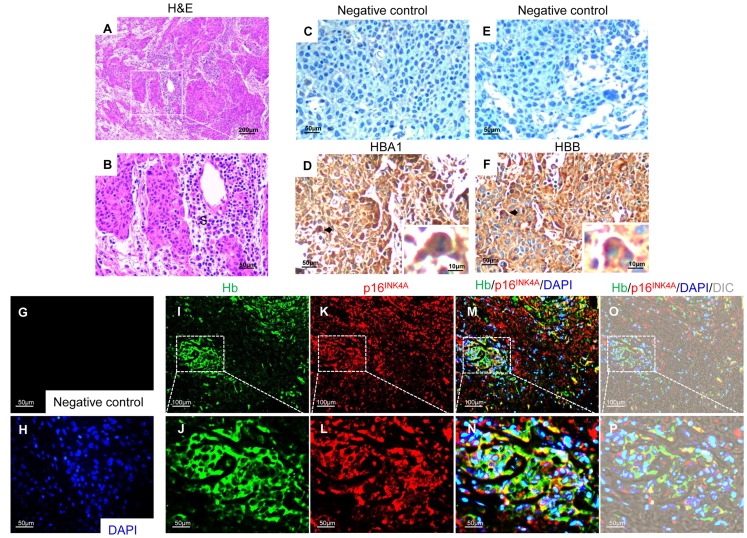
The Hgb protein is expressed in carcinoma tissues of the uterine cervix. Sections from paraffin-embedded human cervical cancer tissues were subjected to either H&E staining or immnunohistochemical analysis with HBA1 and HBB antibodies. Cervical cancer samples showed well-differentiated squamous cell carcinoma (**A** and **B**). S, stroma. Cytoplasmic staining of HBA1 and HBB was detected in biopsy samples from uterine cervical tissues (**D** and **F**). A non-specific IgG monoclonal antibody diluted with PBS was used as a negative control (**C** and **E**). Double-immunostaining against p16^INK4A^, a specific marker of cervical cancer, demonstrated that Hgb was expressed in cervical cancer cells (**I**, **K**, **M** and **O**). White dashed boxes were digitally magnified (**J**, **L**, **N** and **P**). Immunofluorescence without primary antibody revealed negligible signals in cervical cancer cells (**G** and **H**).

The expression of Hgb at the protein level was examined using commercial antibodies against highly purified human Hgb. Because of the high homology among globin family proteins, the specificity of the antibody for the detection of the HBA1 and HBB chains was verified by transfecting human embryonic kidney HEK293 cells with expression vectors carrying EGFP-tagged globin isoforms (Hba-a1, Hbb-b1, Hba-x, Hbb-y, Hba-z), which showed no cross-reaction with other globins in immunostaining experiments (Fig. S1). To avoid cross-reactivity with blood cells, cervical cancer tissues were extensively washed in PBS before fixation. Hematoxylin and eosin (H&E) staining of cervical cancer tissue sections showed well- differentiated squamous cell carcinoma (Fig. 2A and B). In cancerous tissues, HBA1 and HBB showed a diffuse cytoplasmic staining pattern (Fig. 2D and F). A non-specific IgG monoclonal antibody diluted with PBS was used as a negative control (Fig. 2C and E).

The expression of the Hgb protein in uterine carcinoma tissues was confirmed by immunohistochemistry using a specific antibody against Hgb, which showed distinct patterns of diffuse cytoplasmic staining in cervical cancer tissues (Fig. 2I). Omission of the primary antibody in the same immunostaining system served as a negative control (Fig. 2G and H). Double-immunostaining using polyclonal antibodies with cross-reactivity against HBA1 and HBB and an antibody specific for p16^INK4A^, a specific marker of cervical cancer used for cytology and histological diagnosis [28], [29], showed the co-localization of Hgb with p16^INK4A^ in cervical cancer tissues (Fig. 2I–P). These results confirmed that the Hgb protein is expressed in uterine cervical cancer cells.

### Detection of Hgb mRNA and Protein in Cultured Cervical Cancer Cells

**Figure 3:**
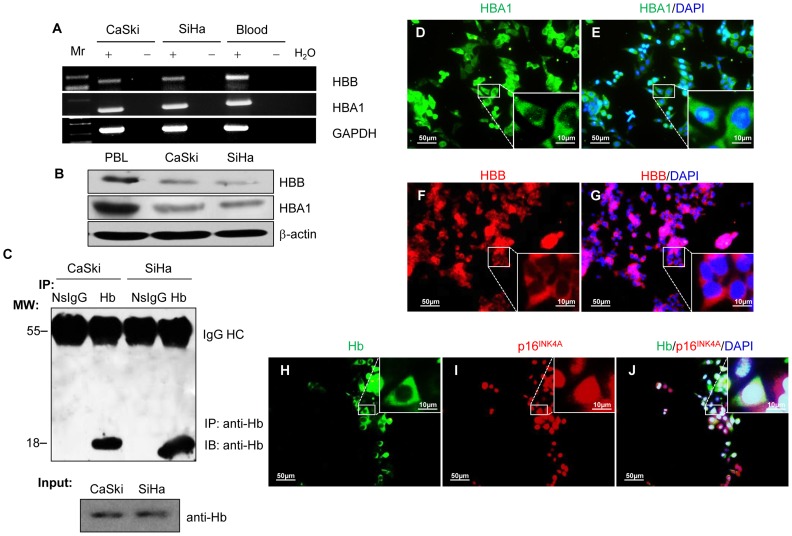
Expression of HBA1 and HBB in cultured cervical cancer cells. (**A**) RT-PCR amplification of HBA1 and HBB from SiHa, CaSki cells and human blood RNA. The HBA1 and HBB transcripts were clearly amplified from the human cervical cancer cell lines, SiHa and CaSki. RNA extracted from blood was used as a positive control and GAPDH was detected as a loading control. Amplicon identities were confirmed by sequencing. (**B**) HBA1 and HBB were analyzed by western blotting. Peripheral blood leukocytes (PBL) were used as a positive control and β-actin was detected as a loading control. (**C**) Cell lysates from SiHa and CaSki cells were immunoprecipitated and immunoblotted with the indicated antibodies. A clear band of 17 kDa was enriched from SiHa and CaSki lysates by immunoprecipitation using an anti-human Hgb antibody. Non-specific IgG was used as immunoprecipitation control. Cytoplasmic staining of HBA1 and HBB was detected in cervical cancer cells (**D** and **E**, **F** and **G**). Double-immunostaining with p16^INK4A^, a specific marker of cervical cancer for cytology and histological diagnosis, demonstrated that Hgb was expressed by cervical cancer cells (**H–J**). Inserts are magnified images of selected areas (small squares).

The expression of Hgb was examined at the mRNA and protein levels using the SiHa and CaSki human cervical carcinoma cell lines cultured *in vitro* to verify our *in vivo* findings of Hgb expression in cervical cancer cells. RT-PCR analysis using human blood cell RNA as a positive control showed the presence of the mRNA for the HBA1 and HBB chains of human Hgb in cultured cervical cancer cells (Fig. 3A). Sequencing of the PCR products showed that they matched 100% with HBA1 (NM_000558) and HBB (NM_000518) mRNA sequences. Consistent with previous studies in alveolar cells and hepatocytes [12], [17], the expression of HBA1 was approximately 9.6 fold higher than that of HBB by qRT-PCR (data not shown). Western blot analysis using specific antibodies against HBA1 and HBB showed low levels of Hgb protein expression in the cell lines analyzed. Protein extracted from peripheral blood leukocytes (PBL) was used as a positive control (Fig. 3B). Immunoprecipitation revealed a clear band of 17-kDa that was specifically enriched from cell lysates (Fig. 3C). Taking advantage of commercial andibodies produced against human HBA1 and HBB, immunofluorescence analysis was performed to examine the localization of the Hgb protein in SiHa cells, which showed a similar cytoplasmic staining pattern of the HBA1 and HBB forms as that of cervical cancer tissues (Fig. 3D–G). Double-immunostaining revealed that Hgb was co-localized with the cervical cancer marker p16^INK4A^ (Fig. 3H–J). Similar results were obtained in another cervical cancer cell line, CaSki (Fig. S2), confirming the expression of Hgb in cultured cervical cancer cells. Notably, the two cell types expressed more HBA1 than HBB at the mRNA and protein levels. As Hgb is likely to act as heterotetramer of two different subunits, we took advantage of co-immunoprecipitation experiments to interrogate whether HBA1 and HBB expression in cervical cancer are able to form heterodimers. As can be seen in Fig. S3, the weak presence of endogenous HBA1/HBB heterodimers was confirmed by co-immunoprecipitation experiments.

### Up-regulation of HBA1 and HBB Expression in Cervical Cancer Cells by Oxidative Stress

**Figure 4:**
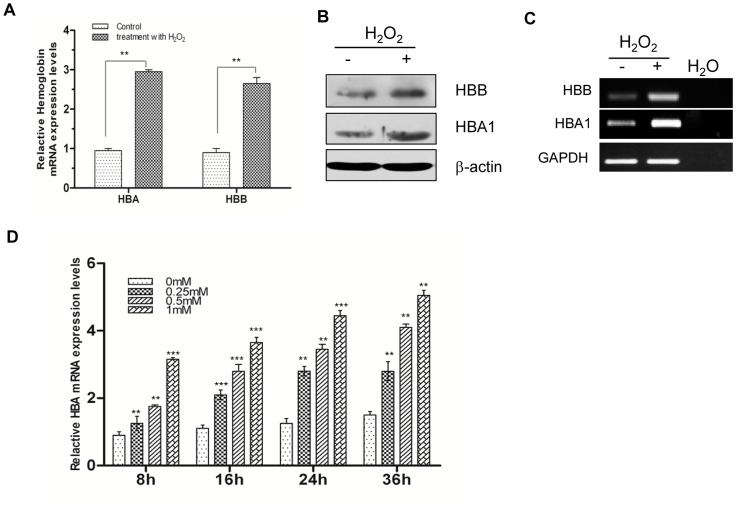
Up-regulation of Hgb expression by oxidative stress. (**A**) HBA1 and HBB mRNA expression in SiHa cells was induced by H_2_O_2_ treatment. SiHa cells were treated with H_2_O_2_ (1 mM) for 24 h and Hgb mRNA levels were determined by qRT-PCR. Hgb levels in controls were normalized to 1. Data were expressed as mean ± SD (n = 3).^ **^ *P*<0.01. (**B**) HBA1 and HBB protein expression was induced by H_2_O_2_ treatment. Whole cell lysates obtained from SiHa cells treated with or without H_2_O_2_ (1 mM, 48 h) were analyzed for HBA1 and HBB levels. β-actin was used as a loading control. (**C**) HBA1 and HBB mRNAs were up-regulated by H_2_O_2_ treatment in HEK293 cells. HEK293 cells were treated with H_2_O_2_ (1 mM, 36 h). Reverse transcription PCR products were separated on 2% agarose gels and visualized with ethidium bromide. GAPDH was used as a loading control. (**D**) Dose and time dependency of the response of SiHa cells to H_2_O_2_ treatment. SiHa cells were treated with a series of concentrations (0, 0.25, 0.5 and 1 mM) of H_2_O_2_ for 8, 16, 24 or 36 h. Relative HBA1 mRNA level was determined by qRT-PCR. Data were expressed as mean ± SD (n = 3). Values were normalized to the HBA1 level in the control (0 mM, 8 h), which was set to 1. One-way ANOVA test for multiple comparisons was used to analyze the differences between treatments. ^**^ *P*<0.01; ^***^ *P*<0.001, when compared to 0 mM treatment.

To determine whether the function of Hgb in nonerythrocytes is different from its known role as oxygen carrier, we examined the expression of the protein in SiHa and CaSki cells in response to oxidative stress based on prior evidence of the antioxidant properties of Hgb. Treatment of SiHa cells with H_2_O_2_ increased the expression of HBA1 and HBB at the mRNA and protein levels (Figs. 4A and B). Similar results were obtained in another cervical cancer cell line, CaSki (Fig. S4). The induction of Hgb by H_2_O_2_ was confirmed in HEK293 cells (Fig. 4C), suggesting that Hgb expression may be up-regulated by oxidative stress in different cell types.

To examine the possible dose and time dependency of the H_2_O_2_-induced up-regulation of Hgb, SiHa cells were treated with different H_2_O_2_ concentrations for various time periods and analyzed by standard qRT-PCR. As seen in Fig. 4D, H_2_O_2_ increased Hgb mRNA levels in a dose- and time-dependent manner. The effect of oxidative stress on the expression of GATA-1 and KLF1, which are transcription factors that regulate Hgb expression during erythropoiesis, was examined. Consistent with a previous study in hepatocytes [17], the up-regulation of Hgb by H_2_O_2_ was not mediated by GATA-1 and KLF1, suggesting that a different underlying mechanism may be involved (Fig. S5).

### Attenuation of ROS Generation and Apoptosis Induction by HBA1/HBB or HBA1, but not HBA1^H88R^/HBB^H93R^ Overexpression in Cervical Cancer Cells

**Figure 5:**
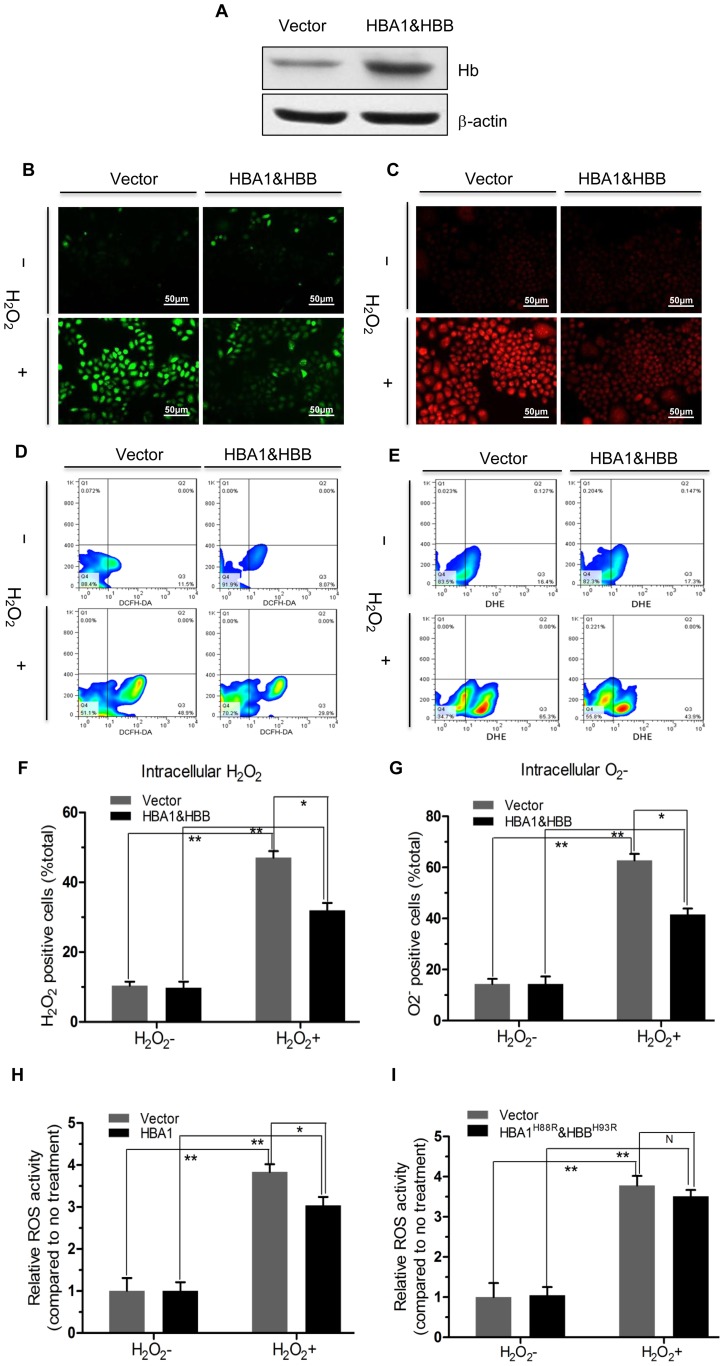
Effect of HBA1/HBB, HBA1 and HBA1^H88R^/HBB^H93R^ overexpression on H_2_O_2_ levels and superoxide anion in SiHa cells undergoing oxidative stress. (**A**) Whole cell lysates from SiHa cells transfected with a control plasmid or HBA1 and HBB expression plasmids were analyzed with an anti-Hb antibody to confirm the overexpression of globin proteins. Control and HBA1/HBB- overexpressing cells were stained with fluorogenic probes to detect intracellular H_2_O_2_ and superoxide anion and exposed to extracellular H_2_O_2_ (1 mM) for 10 min. Immunostaining confirmed that the intracellular generation of H_2_O_2_ (**B**) and superoxide anion (**C**) was suppressed in HBA1/HBB-overexpressing cells. Flow cytometry analysis confirmed these results (**D** and **E**) and showed that the intracellular levels of H_2_O_2_ and superoxide anion were higher in cells exposed to extracellular stimuli than in untreated cells (**B** and **C**). Quantitative analyses confirmed that the intracellular generation of H_2_O_2_ (**F**) and superoxide anion (**G**) was inhibited in HBA1/HBB-overexpressing cells (black column) more than in control cells (gray column; *P*<0.05). Quantitative analyses confirmed that the intracellular generation of ROS was reduced in HBA1-overexpressing cells (black column, **H**), but not in HBA1^H88R^/HBB^H93R^-overexpressing cells (black column, **I**) more than in control vector-overexpressing cells (gray column). *P<0.05; **P<0.01; ^N^ *P*>0.05.

To investigate the biological role of Hgb in cervical cancer cells, HBA1 and HBB were overexpressed by transient transfection of SiHa cells. Increased expression of HBA1 and HBB was confirmed by immunoblotting (Fig. 5A). Based on a previous study showing that certain oxidative stress inducible genes have antioxidative functions [30], we hypothesized that Hgb may have antioxidant properties in cervical cancer cells in response to oxidative stress. We therefore examined the intracellular levels of ROS in cells treated with H_2_O_2_ using fluorogenic probes and the superoxide anion. Ectopic expression of HBA1/HBB in SiHa cells treated with exogenous H_2_O_2_ suppressed the intracellular production of H_2_O_2_ and superoxide anion as determined by immunofluorescence analysis (Fig. 5B and C). Quantification of ROS production by flow cytometry showed that the fluorescence intensity in cells overexpressing HBA1/HBB was significantly lower than in cells transduced with control lentivirus (Fig. 5D and E). Percentage per total cell count with high levels of each ROS in cells overexpressing HBA1/HBB was significantly reduced compared with transfection with the empty vector as a control (*P*<0.05, n = 3 for each group; Fig. 5F and G). Similar results were observed in CaSki cells (data not shown). The fact that the expression level of HBA1 in cervical cancer cell lines is higher than that of HBB suggests that multiple bioactive molecules, not only the heterodimer forms but also monomer or homodimer forms of the HBA1 protein may be present at the same time in cervical cancer cells. In support of this notion, SiHa cells were merely transfected with HBA1 and then treated with exogenous H_2_O_2_. Consistent with a recent study [31], percentage per total cell count with intracellular levels of ROS in cells overexpressing HBA1 was significantly lower than that in cells transfected with the control vector (Fig. 5H). To discard any generalized or unselective effect due to the non-specific overexpression of protiens, we generated a cell line that expresses an inactive form of Hgb carrying an aminoacid change at the His responsible for coordinating the hemo prosthetic group (HBA1^H88R^ [31] and HBB^H93R^, Fig. S6). As can be seen in Fig. 5I, the intracellular ROS levels in cells overexpressing HBA1^H88R^/HBB^H93R^ were not significantly reduced, compared with that in cells transfected with empty vector, suggesting that heme-binding activity is required for Hgb antioxidative function.

**Figure 6:**
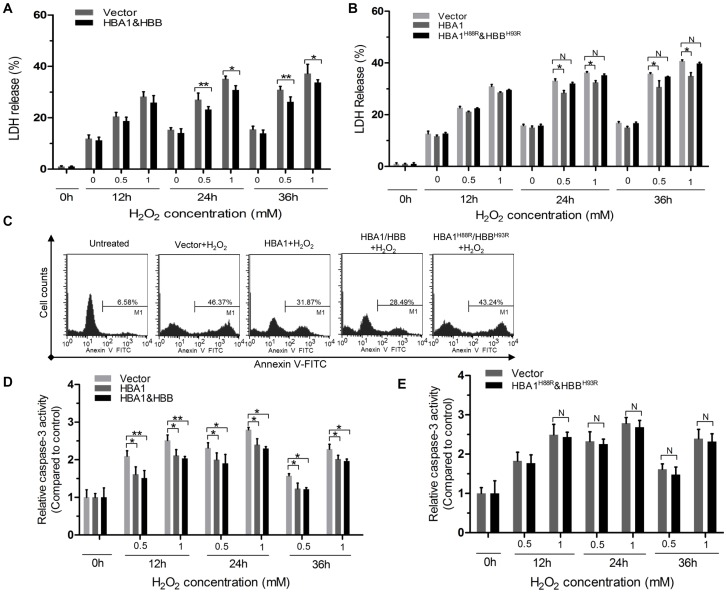
Either HBA1/HBB or HBA1, but not HBA1^H88R^/HBB^H93R^ overexpression protects cells against oxidative stress-induced damage. (**A**) The viability of SiHa cells treated with 0, 0.5, and 1 mM H_2_O_2_ for 12, 24 and 36 h as extracellular oxidative stress was evaluated by the LDH release assay. SiHa cells transfected with the HBA1/HBB-expressing vectors (black column) showed increased resistance against H_2_O_2_-induced cell compared to cells transfected with the control vector (gray column). Similar experiments were preformed in both HBA1-overexpression cells and HBA1^H88R^/HBB^H93R^-overexpression cells (**B**). SiHa cells were transfected with the indicated plasmids. Forty-eight hours later, cells were treated with 1 mM H_2_O_2_ or left untreated for 22 h. The percentage of apoptotic cells was monitored by Annexin V staining followed by FACS analysis (**C**). SiHa cells were transfected with the indicated plasmids and forty hour later, were treated as in (A), Caspase-3 activity in different group was measured using specific caspase substrate AcDEVD-pNA as described under MATERIALs AND METHODs. Values of the absorption were expressed as control which was set as 1 (**D** and **E**). All experiments were performed in triplicate and were repeated at least three times, and the results are expressed as means ± S.E.M.*P<0.05; **P<0.01; ^N^ *P*>0.05.

A lactate dehydrogenase (LDH) cytotoxicity assay, which measures the release of LDH into the culture medium, was performed to examine cell viability under oxidative stress. SiHa cells overexpressing HBA1 and HBB showed LDH release rates of 22.1±2.2% and 32.5±3.4% in response to treatment with 0.5 mM and 1 mM H_2_O_2_ for 24 h, respectively, compared to 29.6±3.1% and 34.6±2.1%, respectively, in cells transfected with empty vector (*P*<0.01 and *P*<0.05 for 0.5 and 1 mM H_2_O_2_, respectively; n = 3 for each group) (Fig. 6A). SiHa cell viability was also improved by HBA1 and HBB overexpression when cells were treated with 0.5 mM and 1 mM H_2_O_2_ for 36 h (*P*<0.01). Similar results were obtained in HBA1-overexpression SiHa cells (Fig. 6B). We then investigated whether the function of Hgb to improve cell viability depends on its heme-binding activity, using the HBA1^H88R^/HBB^H93R^ mutants, which are unable to bind heme but preserve chain conformation. As can be seen in Fig. 6B, mutation of the His responsible for coordinating the heme prosthetic group (HBA1^H88R^ and HBB^H93R^) of HgbA impairs its ability to improve cell viability.

ROS (superoxide, hydrogen peroxide, and hydroxyl radicals) are potent intracellular oxidants and inducers of oxidative damage, which have been proposed as critical regulators of apoptosis [32] and treatments with antioxidants protect cells from apoptosis [33]. Given that the presence of Hgb (either HBA1 or HBA1/HBB) leads to reduce production of interacellular ROS, it is conceivable that such cells retain the ability to protect themselves from oxidative insults. Thus, we raised the question whether Hgb-mediated protection against cell death in SiHa cells was simply because of reduced intracellular accumulation of ROS. We explored this possibility by determining the effect of wild type HBA1/HBB, HBA1, and mutants HBA1^H88R^/HBB^H93R^-overexpression upon H_2_O_2_-induced apoptosis by flow cytometric analysis using SiHa cells. As shown in Fig. 6C, the results showed that the population of the apoptotic cells induced by H_2_O_2_ was inhibited in both HBA1 and HBA1/HBB-overexpression cells, but not in HBA1^H88R^/HBB^H93R^-overexpression cells. Activation of a cysteine protease, called caspase-3 and the generation of ROS were considered key steps in the induction of cell death [34] and it is known that ROS generation in mitochondria activates caspase-3 via cooperation of cytochrome *c*, Apaf-1 and caspase-9 [35]. We next investigated the effect of Hgb on caspase-3 activation. Consistent with the Annexin V binding assay, we found that the increasing activation of caspase-3 caused by 0.5 mM and 1 mM H_2_O_2_ after incubating for 12, 24 and 36 h, respectively, could be significantly reduced in both HBA1 and HBA1/HBB-overexpression cells, compared with the empty vector (Fig. 6D). In contrast, ectopic expression of HBA1^H88R^/HBB^H93R^ in SiHa cells was not effective in preventing H_2_O_2_-induced caspase-3 activation (Fig. 6E), suggesting that heme-binding activity is required for its function. In summary, these results suggested that both HBA1 and HBA1/HBB overexpression may have an antioxidant effect via scavenging of ROS, thus protecting cervical cancer cells against oxidative stress-induced damage.

## Discussion

In the present study, we showed that Hgb expression was elevated in cervical cancer tissues compared with normal cervix tissues. This is the first evidence that the α- and β-globin chains of Hgb are expressed in cancer cells of the uterine cervix. We used the cervical carcinoma cell lines SiHa and CaSki to examine Hgb-α and Hgb-β mRNA and protein expression using three different approaches: RT-PCR, immunostaining and western blot analysis. Furthermore, Hgb attenuated hydrogen peroxide induced oxidative stress in cervical cancer cells, acting as an antioxidant.

The long-standing notion that the Hgb is expressed only in the cells of erythroid lineage has been challenged in recent studies showing Hgb expression in nonerythrocytes, including neurons [7], [8], [9], retinal cells [10], [11], alveolar epithelial cells [12], [13], [14], endometrium [15], rat kidney mesangial cells [16], hepatocytes [17] and macrophages [18]. The main function of Hgb in erythrocytes is to transport oxygen from the lung to the tissues and to transport carbon dioxide from the tissues to the lung. The biological function of Hgb in nonerythroid cells remains to be elucidated and its physiological function is still a matter of debate. Hgb overexpression in a murine dopaminergic cell line, MN9D, altered the expression of various genes involved in oxygen homeostasis and mitochondrial oxidative phosphorylation, suggesting that Hgb may function as an oxygen storage molecule in neurons [9]. Hgb overexpression in the rat renal mesangial cell line SV40-MES13 and the human hepatocellular cancer cell line HepG2 reduced hydrogen peroxide- induced oxidative stress, suggesting that Hgb functions as an antioxidant [16], [17].

Although the function of Hgb in cancer cells of the uterine cervix has not been investigated in detail, here we have provided some interesting cues by using a well-accepted *in vitro* model of human cervical cancer, SiHa and CaSki cells, to investigate the role of Hgb in cervical cancer cells.

Erythropoiesis is a complex multistep process and several transcription factors are involved in erythroid differentiation [27]. The expression of Hgb is well coordinated and tightly controlled at the late stages of erythropoiesis. However, the α- and β-globin clusters are located on different chromosomes and have diverged into different genomic contexts. Individual Hgb genes contain elements that result in their temporal expression during vertebrate development [36]. Because it is traditional dogma that Hgb genes are expressed solely by erythroid tissue in vertebrates, our results raise important questions in the fields of hematology, genetics, pathology and development. Furthermore, the regulation of Hgb expression in cancer cells is not fully understood. Our results showed significantly lower amounts of globin polypeptides in cancer cells than those reported in erythrocytes, suggesting the possibility that globin genes may not be similarly regulated in cancer cells and erythroid cells. In a recent study, Hgb transcription was increased by hypoxia through the up-regulation of the transcription of GATA-1 in alveolar epithelial cells [14]. In murine macrophages, treatment with LPS and IFN-γ led to the activation of the β-globin gene [18]. Our results showed that oxidative stress had no significant effect on the expression levels of GATA-1 and KLF1 (Fig. S4), suggesting that in cervical cancer cells, the induction of Hgb expression by oxidative stress is mediated by a different mechanism. Further studies are needed to elucidate the mechanism underlying the induction of Hgb expression by oxidative stress in cervical cancer.

The role of oxidative stress in carcinogenesis has been studied extensively in recent years, and several antioxidants have been investigated in this context [37], [38]. The levels of ROS are controlled by antioxidant enzymes, such as superoxide dismutase (SOD) and glutathione peroxidase (GSH-Px). Under normal physiological conditions, cellular redox homeostasis is maintained by the balance between ROS formation and the activity of antioxidant enzymes. However, this balance can be easily disturbed, leading to oxidative stress and damage to the cell [39]. A growing body of studies has indicated that the oxidant/antioxidant status of patients can influence tumor growth and proliferative activity and subsequently overall survival and risk of recurrence after surgery [40], [41]. A previous study has shown that Hgb detoxify highly oxidizing radicals yielding the respective ferric states, which are not toxic [42]. Furthermore, Hgb was shown to remove H_2_O_2_ more efficiently than the glutathione peroxidase-glutathione reductase system [43]. In addition, a recent study showed that Hgb functions as an antioxidative peroxidase, attenuating H_2_O_2_-induced oxidative stress [22]. Notably, Hgb was found to promote the proliferation of colon cancer cells [44]. In the present study, mRNA transcripts corresponding to the two globin subunits of adult Hgb were detected in several solid tumor cell lines (Fig. S7). Although the exact role of Hgb in carcinogenesis has yet to be determined, the expression of Hgb in cancer cells may have important implications in the pathology of solid tumors because of the functions inherent to the structure of the Hgb molecule, including gas exchange, and protection against oxidative and nitrosative stress [45], [46]. In addition, Hgb might also function as an antioxidative peroxidase [22], attenuating hydrogen peroxide induced oxidative stress in cancer cells. However, the physiological implications of the present study are mainly based in overexpression experiments and thus their significance is limited. Additional experiments, including loss of function analysis, are required to support a role for HgbA in cervical cancer cell redox regulation. Furthermore, the microarray studies of gene expression analysis are necessary to elucidate the role of Hgb in the development and growth of solid tumors, and to determine whether Hgb may function in cancer cells as a regulator in oxygen homeostasis, mitochondrial function and oxidative defenses molecule for the maintenance of redox homeostasis under anoxic conditions.

In conclusion, our results show, for the first time, that Hgb is expressed by cervical carcinoma cells and plays a cytoprotective role against oxidative insults. These findings suggest that Hgb may act as an endogenous antioxidant defense protein in cervical cancer cells, and provide important information for the understanding of the pathogenesis and progression of different cancers under conditions of oxidative stress associated with radiotherapy and chemotherapy.

## Materials and Methods

### Patients and Clinical Specimens

This study was approved by the Institutional Ethics Committee of the Chinese PLA General Hospital and written consent was obtained from all participating patients. Squamous cell cervical cancer biopsy specimens were surgically resected from 20 patients who were undergoing examination under general anesthesia as part of their pretreatment evaluation for cervical cancer at the General Hospital of PLA (Beijing, China). 10 normal cervical tissue specimens were collected from healthy patients after obtaining informed consent and used as controls in the study. Tissue samples were frozen in liquid nitrogen immediately after resection and used for qRT-PCR and immunostaining. Additional freshly excised tissues were fixed in paraffin, cut into 2 to 4 µm thick sections and stained with hematoxylin and eosin (H&E) or used for immunohistochemistry.

### Cell Culture and Transfection

The human cervical carcinoma cell lines SiHa and CaSki were obtained from the American Type Culture Collection (ATCC, Rockville, MD, USA) and cultured in Minimum Essential Medium (MEM) and RPMI1640, respectively, supplemented with 10% fetal bovine serum (FBS) (Gibco BRL, Life Technologies, Inc., Maryland, USA), 100 IU ml^−1^ penicillin and 10 µg ml^−1^ streptomycin according to the instructions provided. Cultures were maintained at 37°C in a humidified atmosphere with 95% air and 5% CO_2_. Cell transfection was carried out as described previously [47]. Forty eight hours after transfection, cells were harvested for western blot analysis or used for flow cytometry.

### Microarray Analysis

Previously published microarray data including 33 cervical cancer and 24 normal cervix samples was analyzed to identify differentially expressed genes in cervical cancer tumorigenesis [48]. The clinical characteristics of the cervical cancer patients were shown in Table 2. The original dataset has been uploaded to Gene Expression Omnibus (GEO; website: http://www.ncbi.nlm.nih.gov/sites/GDSbrowser). GEO accession number for the dataset is GSE9750.

**Table 2:** Clinicopathological characteristics of the cervical cancer patients.

Patient characteristics	Number
**Age**	
≤50 years	19
>50 years	12
NA	2
**FIGO stage**	
Stage I	5
Stage II, III, IV	22
NA	6
**HPV type**	
Negative	2
Positive of any HPV type (total)	31
HPV16	19
HPV45	3
HPV18	3
HPV16+HPV18	1
HPV18+HPV45	2
HPV16+HPV31+HPV45	2
HPV16+HPV18+HPV31+HPV45	1

FIGO: International Federation of Gynecology and Obstetrics; HPV: Human papillomavirus; NA, Not available.

### Quantitative Real Time PCR (qRT-PCR)

Total RNA was isolated using the TRIzol reagent (Invitrogen, Carlsbad, CA) according to the manufacturer’s instructions, and treated with RNase-free DNase (Cat. #M6101, Promega, Madison, WI) to eliminate contaminating DNA. cDNA was prepared from 1 to 2 µg of total RNA using PrimeScript™ reverse transcriptase (Takara Shuzo Co., Tokyo, Japan) and subjected to qRT-PCR using the SYBR Green PCR Reagents Kit (Bio-Rad). Glyceraldehyde-3-phosphate dehydrogenase (GAPDH) was used as internal control. Relative gene expression was calculated as described previously [49]. The primers used for qRT-PCR were as follows: HBA1 sense, 5′-GTC GGC GCG CAC GCT GGC GAG T-3′, HBA1 antisense, 5′-GCG CGT TGG GCA TGT CGT CCA C-3′; HBB sense, 5′-GGA CCC AGA GGT TCT TTG AGT C-3′, HBB antisense, 5′-GGC CAG CAC ACA GAC CAG CAC G-3′; GATA-1 sense, 5′-CCC TCA ATT CAG CAG CCT ATT CC-3′, GATA-1 antisense, 5′-TTT CCA GAT GCC TTG CGG TTT-3′; KLF-1 sense, 5′-TCG GAG GAT CAC TCG GGT TGG-3′, KLF-1 antisense, 5′-ACG CCG CAG GCA CTG AAA GC-3′; SCL/TAL1 sense, 5′-GAG CCG GAT GCC TTC CCT ATG TT-3′, SCL/TAL1 antisense, 5′-CCT CCT GGT CAT TGA GCA GCT TGG-3′; GAPDH sense, 5′- AGC CTC AAG ATC ATC AGC AAT G -3′, GAPDH antisense, 5′-ATG GAC TGT GGT CAT GAG TCC TT-3′.

### Immunoblotting and Co-immunoprecipitation

Coimmunoprecipitation and immunoblotting were performed as reported previously [47], [49]. For immunoprecipitation and coimmunoprecipitation, cells were lysed in immunoprecipitation buffer [300 mM NaCl, 50 mM Tris (pH 7.5), 1% Nonidet P-40, 10% glycerol], supplemented with a complete EDTA-free protease inhibitor mixture for 30 min on ice. Lysates were cleared by centrifugation at 12,000× g for 20 min and incubated with the indicated antibodies or the corresponding controls. Immunoprecipitation was performed at 4°C overnight. After washing in immunoprecipitation buffer, precipitated proteins were eluted with 2× sodium dodecyl sulfate (SDS) sample buffer, boiled, and analyzed by western blotting. For immunoblotting, whole cell lysates from SiHa and CaSki cells were prepared using SDS lysis buffer. Protein concentration was measured using a modified Bradford method. Total cell lysate samples containing 60 µg of protein along with a protein ladder (SM0671, Fermentas) were subjected to SDS-PAGE (NP0301, Invitrogen, Carlsbad, CA) and transferred onto methanol-activated polyvinylidene fluoride membranes (LC2002, Invitrogen, Carlsbad, CA). Membranes were blocked with 5% skim milk for 60 min at room temperature, incubated with primary antibody overnight at 4°C and then washed three times, followed by incubation with horseradish peroxidase-conjugated secondary antibody for 45 min. After washing three times, the protein bands were visualized using ECL reagents. Antibodies specific to Hba1, Hbb, and β-actin were purchased from Santa Cruz Biotechnology.

### Immunohistochemistry

For immunohistochemical studies, tissues were dissected and fixed flat between wet filter papers overnight in 4% paraformaldehyde. Tissue specimens were routinely embedded in paraffin wax, cut into 4 µm sections and mounted on organosilane coated slides. The 4 µm paraffin sections were dewaxed in xylene and retrieved by boiling in a 10 mM citrate buffer (pH 6.0) for 20 min. Endogenous peroxidases were blocked in 0.3% H2O2 in methanol for 30 min. To prevent nonspecific binding, sections were blocked in PBS containing 3% bovine serum albumin (BSA) and 0.1% Nonidet P-40 (blocking buffer) for 10 min at room temperature, and then incubated with primary antibodies against HBA1 and HBB (Santa Cruz Biotechnology, USA) diluted in blocking buffer overnight at 4°C. Negative controls were incubated in blocking buffer without primary antibody, treated with the secondary antibody (biotin-labeled anti-rabbit IgG goat antibody) for 45 min at room temperature, washed with PBS, and finally incubated with peroxidase-labeled streptavidin for 50 min. After washing in PBS, sections were developed with a 3,3′-diaminobenzidine tetrahydrochloride substrate (DAKO), which resulted in the production of a brown stain. The sections were lightly counterstained with Mayer’s hematoxylin, dehydrated through a graded ethanol series and xylene, and mounted.

### Immunofluorescence Microscopy

Immunostaining was performed as described previously [47]. Cryostat tissue sections (9 µm thick) and cultured cells fixed on cover slips were incubated with specific antibodies against human HBA1, HBB, Hgb and p16^INK4A^ (Abcam, Cambridge, U.K) using standard immunohistochemical procedures. Alexa Fluor-488, or -594 (Invitrogen, Carlsbad, CA) antibodies were used for protein detection. Nuclei were counterstained with 4,6-diamidino-2-phenylindole (DAPI). Immunofluorescence was visualized with a confocal microscope (Leica TCS SP2).

### Construction of Vectors and Lentivirus Production

Total RNA from human peripheral blood was reverse-transcribed, and the resulting cDNA was amplified by PCR using oligonucleotide primers designed based on the open reading frames coding for HBA1 (NM_000558) and HBB (NM_000518). The following PCR primer pairs were used: HBA1 sense, 5′-GGA AGA TCT GCC ACC ATG GTG CTG TCT CCT GCC GAC AA-3′ and antisense, 5′-ACG CGT CGA CAC GGT ATT TGG AGG TCA GCA-3′; HBB sense, 5′-GGA AGA TCT GCC ACC ATG GTG CAT CTG ACT CCT GAG GA-3′, and antisense, 5′-ACG CGT CGA CGT GAT ACT TGT GGG CCA GGG-3′, both of which included the Kozak sequence. PCR fragments were subcloned into pGEM-T Easy vectors to produce pGEM-T/HBA1 and pGEM-T/HBB, which were digested with Bgl II/Sal I and inserted into the same region of the multiple cloning sites in pEGFP-N1, generating pEGFP-N1/HBA1 and pEGFP-N1/HBB. Two overlapping PCR products were synthesized using the 5′-CCG AAG CTT GTG CGC GCG CAG G-3′ and 5′- GCC CTG AGC GAC CTG CGC GCG C-3′ and the 5′-CGT GCA GCT TGT CAC AGC GCA GC-3′ and 5′-CAC ACT GAG TGA GCT GCG CTG TG-3′, respectively. The underlined bases were those used to create the desired mutations His88 (HBA1) → Arg and His93 (HBB) → Arg. PCR amplifications were performed as described above to produce the recombinant HBA1 gene containing the H88R mutation and the HBB gene containing H93R mutation. The recombinant HBA1 and HBB genes with the termination codon were excised as the Bgl II/Sal I fragment and used to replace the Bgl II/Sal I fragment of pEGFP-N1. The recombinant plasmid with desired mutations was identified directly by DNA sequence analysis and the base change encoding the His88 (HBA1) → Arg and His93 (HBB) → Arg was only mutation of the HBA1 and HBB gene, respectively (Fig. S6). Other globin chains (Hba-x, Hba-y, Hba-z) were also cloned into the pEGFP-N1 vector. Constructs were verified by DNA sequencing and purified using a plasmid purification kit (Qiagen, Hilden, Germany). Lentiviral vectors (pWPT-v5) containing the cDNAs of HBA1 and HBB were transfected using the ViraPower Lentiviral Packaging Mix (Invitrogen) in a HEK293-FT packaging cell line. Viral supernatants were harvested 48 h after transfection and ultracentrifuged to concentrate the virus. Equal amounts of each virus were used for SiHa transduction in the presence of 8 µg ml^−1^ polybrene.

### Detection of Intracellular ROS and Superoxide Anion

Oxidative stress was assessed by measuring the intracellular levels of reactive oxygen species (ROS) generated after exposure of cells to H_2_O_2_ using fluorogenic probes specific for H2O2 and superoxide anion. After extensive washing in PBS, SiHa and CaSki cells were treated with 10 µM 6-carboxy-2′,7′-dichlorofluorescein diacetate (DCFH-DA, Beyotime, China) or 5 µM dihydroethidium (DHE, Beyotime, China) at 37°C in the dark for 30 min to detect intracellular ROS and superoxide anion, respectively. Cells were washed in PBS and treated with or without 1 mM H_2_O_2_ for 10 min, then harvested by trypsinization. Fluorescence signals were analyzed by microscopy and FACS analysis on a Becton Dickinson FACScan flow cytometer (DCF: excitation wavelength, 488 nm, and emission wavelength, 535 nm; DHE: excitation wavelength, 325 nm, and emission wavelength, 610 nm).

### Cell Viability Assay and Assessment of Apoptotic Cells by Flow Cytometry

To determine the biological significance of cellular Hgb as an antioxidant, SiHa and CaSki cell viability was assessed using the LDH release assay as described previously [50]. Cells stably overexpressing HBA1 and HBB were exposed to 0.5 to 1 mM H_2_O_2_ for 12 to 36 h under serum-free conditions. Percent cell death was determined by the amount of LDH in the medium divided by the total amount of LDH in the medium and the cell lysate. The apoptosis assay using flow cytometry was performed as reported previously [47]. Cells that had been transfected stably with Hgb or Hgb mutants were treated with H_2_O_2_ (1 mM) or left untreated for 22 h. Floating and adherent cells were collected and analyzed using the Annexin V-FITC Apoptosis Detection Kit I (BD Bioscience, CA, USA) in accordance with the manufacturer’s instructions. A FACSCalibur flow cytometer (BD Biosciences) was used. All experiments were performed in triplicate.

### Quantification of Caspase-3 Activity

The activity of caspase-3 was assessed using Caspase-3 Colorimetric Assay Kits (Beyotime, China), which are based on the spectrophotometric detection of the color reporter molecule p-nitroanilide (pNA) after cleavage from the labeled substrate acetyl-Asp-Glu-Val-Asp p-nitroanilide (Ac-DEVD-pNA) as an index. In brief, cells were incubated with the designated dose of H_2_O_2_ for 12 to 36 h.The cells were then washed in PBS and suspended in 100 µl lysis buffer (20 mM HEPES, pH 7.9, 20% glycerol, 200 mM KCl, 0.5 mM EDTA, 0.5% NP-40, 0.5 mM DTT, 1% protease inhibitor cocktail) for 30 min on ice. The cell lysates were centrifuged at 12, 000× g at 4°C for 20 min and then collected, and protein concentration was determined by the Bradford method. Supernatant samples containing 50µg of total protein were used for determination of caspase-3 activity. These are added to each well in 96-well microtiter plates with the DEVD-pNA at 37°C for 2 h. The optical density of each well was measured at 405 nm using a microplate reader. Each plate contained multiple wells of a given experimental condition and multiple control wells. The activity of caspase-3 was expressed in arbitrary absorbance units (absorbance at a wavelength of 405 nm).

### Statistical Analysis

Experimental results were expressed as the mean ± standard error of the mean (S.E.M.). Statistical analysis was performed using Statview 5.0 software. Unpaired Student’s t-test or analysis of variance (ANOVA) followed by Tukey’s post-hoc test were used where applicable to assess significant differences between groups. P values≤0.05 were considered statistically significant.

## Supporting Information

Figure S1 **Antibody specificity test.** HEK293 cells transfected with constructs expressing different globin chains (Hba-a1, Hbb-b1, Hba-x, Hbb-y, Hba-z) were subjected to immunofluorescence analysis using commercial anti-Hba1, anti-Hbb and anti-Hb antibodies (Red). The transfected constructs also expressed EGFP (Green). pEGFP-N1 empty vectors were transfected as controls. Commercial anti-Hbb-a1 and anti-Hbb-b1 antibodies specifically recognized HBA1 and HBB, respectively (**a**). An anti-Hb antibody specifically recognized both HBA1 and HBB chains. No cross-reaction was observed with other globins (**b**). (Magnification: 63x.)
(TIF)

Figure S2 **Double-immunofluorescence studies in CaSki cells showed the localization of the Hgb protein.** A cytoplasmic staining pattern was detected in CaSki cells probed with an anti-Hgb antibody. Double-immunostaining with p16^INK4A^, a marker of cervical cancer cells, confirmed the expression of Hgb in CaSki cells.
(TIF)

Figure S3 **The presence of endogenous HBA1 and HBB heterodimers in cervical cancer SiHa cells.** Immunoprecipitation (IP) of HBA1 (IP anti-HBA1) from SiHa cells co-immunoprecipitated HBB (immunoblot, IB, anti-HBB) demonstrating that the endogenous HBA1 and HBB chains are able to form heterodimers. Reverse coimmunoprecipitation confirm this result (IP anti-HBB, IB, anti-HBA1). Non-specific IgG was used as IP control. Asterisk indicates each immunoprecipitated protein.
(TIF)

Figure S4 **HBA1 and HBB mRNA expression was induced by H_2_O_2_ in CaSki cells.** CaSki cells were treated with or without H_2_O_2_ (0.25, 0.5 mM, 24 h) and harvested for HBA1 and HBB mRNA analyses by RT-PCR. PCR products were separated on 2% agarose gels and visualized with ethidium bromide. GAPDH was used as a loading control.
(TIF)

Figure S5 **Oxidative stress has no significant effect on the expression levels of GATA-1 and KLF1.** Relative GATA-1 and KLF1 mRNA levels determined by qRT-PCR were similar in untreated controls and in SiHa cells treated with H_2_O_2_ (1 mM) for 8, 16, 24, or 36 h. Data represent mean ± SD of three RT-PCR reactions (^N^ *P*>0.05).
(TIF)

Figure S6 **Wild type and mutated Hgb plasmids were verified by sequencing.** Schematic representation of the constructions used for overexpressing wild type (HBA1 and HBB) and mutated (HBA1^H88R^ and HBB^H93R^) Hgb forms in SiHa cells. Black lines represent the CMV-IE promoter, light gray lines represent the HBA1 and HBB genes. All mutation incorporation into the plasmid DNA were verified by sequencing.
(TIF)

Figure S7 **Expression of HBA1 and HBB chains in different solid tumor cell lines.** The expression of HBA1 and HBB mRNA was analyzed by RT-PCR. PCR products were separated on 2% agarose gels and visualized with ethidium bromide. Retrotranscriptase free (RT–) as a negative control, H_2_O, as a system control.
(TIF)
