# Supplementary figures and images for: Characterization of Adult α- and β-Globin Elevated by Hydrogen Peroxide in Cervical Cancer Cells That Play A Cytoprotective Role Against Oxidative Insults
Source: PLoS One. 2013 Jan 17;8(1):e54342. doi: 10.1371/journal.pone.0054342 (PMC3547883; doi:10.1371/journal.pone.0054342)

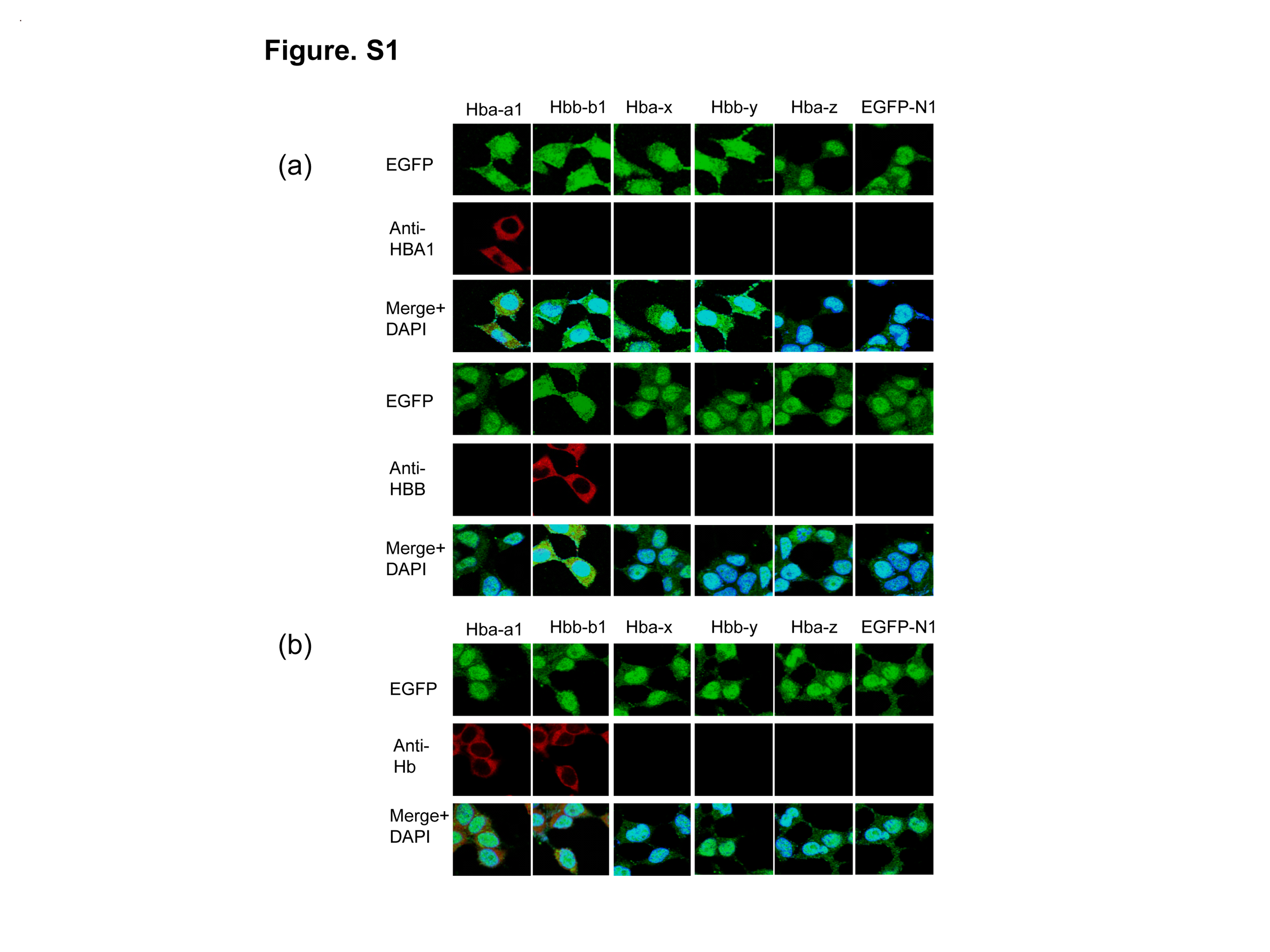

Supplement: Figure S1 — Antibody specificity test. HEK293 cells transfected with constructs expressing different globin chains (Hba-a1, Hbb-b1, Hba-x, Hbb-y, Hba-z) were subjected to immunofluorescence analysis using commercial anti-Hba1, anti-Hbb and anti-Hb antibodies (Red). The transfected constructs also expressed EGFP (Green). pEGFP-N1 empty vectors were transfected as controls. Commercial anti-Hbb-a1 and anti-Hbb-b1 antibodies specifically recognized HBA1 and HBB, respectively (a). An anti-Hb antibody specifically recognized both HBA1 and HBB chains. No cross-reaction was observed with other globins (b). (Magnification: 63x.) (TIF) [file pone.0054342.s001.tif]

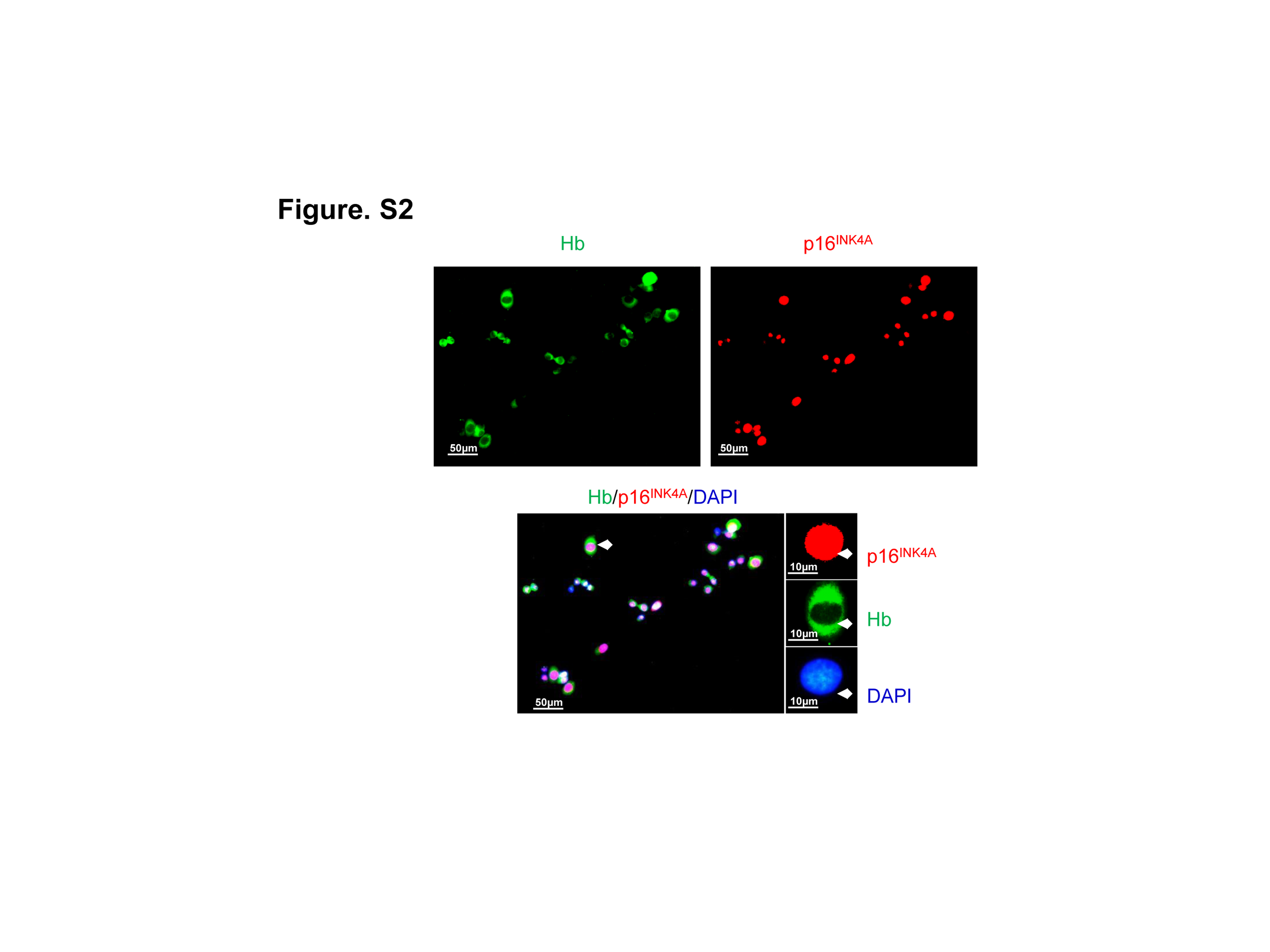

Supplement: Figure S2 — Double-immunofluorescence studies in CaSki cells showed the localization of the Hgb protein. A cytoplasmic staining pattern was detected in CaSki cells probed with an anti-Hgb antibody. Double-immunostaining with p16INK4A, a marker of cervical cancer cells, confirmed the expression of Hgb in CaSki cells. (TIF) [file pone.0054342.s002.tif]

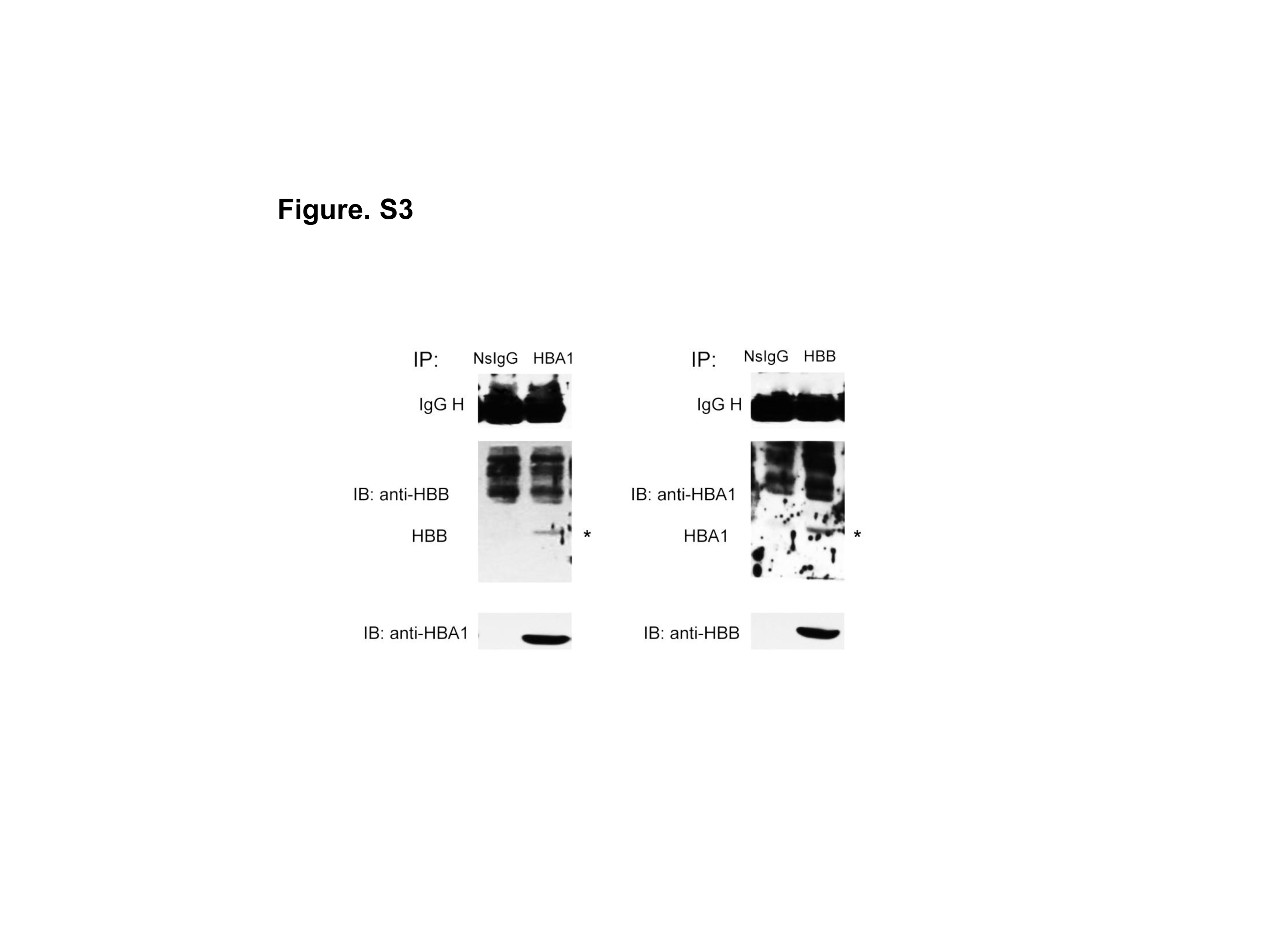

Supplement: Figure S3 — The presence of endogenous HBA1 and HBB heterodimers in cervical cancer SiHa cells. Immunoprecipitation (IP) of HBA1 (IP anti-HBA1) from SiHa cells co-immunoprecipitated HBB (immunoblot, IB, anti-HBB) demonstrating that the endogenous HBA1 and HBB chains are able to form heterodimers. Reverse coimmunoprecipitation confirm this result (IP anti-HBB, IB, anti-HBA1). Non-specific IgG was used as IP control. Asterisk indicates each immunoprecipitated protein. (TIF) [file pone.0054342.s003.tif]

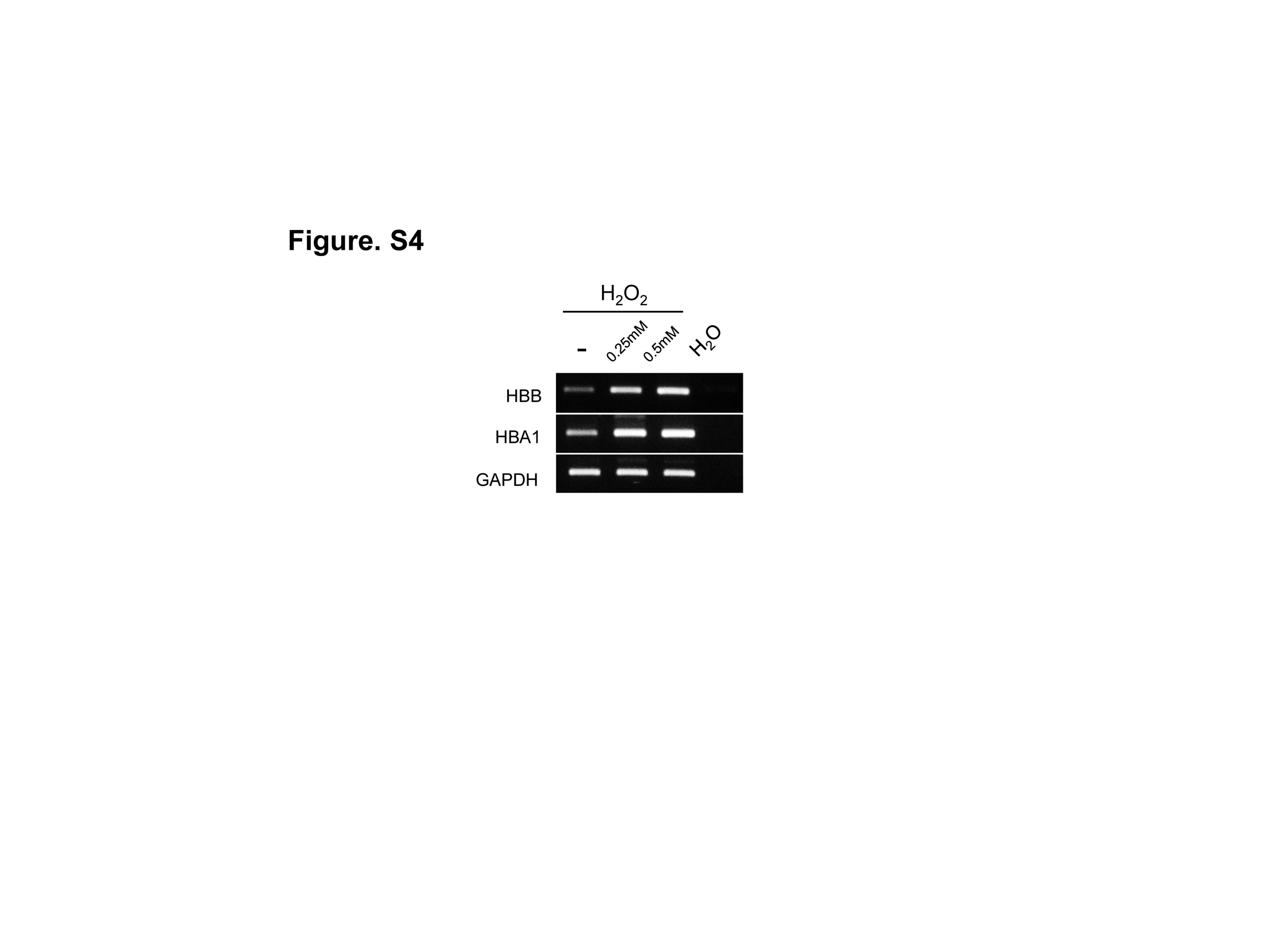

Supplement: Figure S4 — HBA1 and HBB mRNA expression was induced by H2O2 in CaSki cells. CaSki cells were treated with or without H2O2 (0.25, 0.5 mM, 24 h) and harvested for HBA1 and HBB mRNA analyses by RT-PCR. PCR products were separated on 2% agarose gels and visualized with ethidium bromide. GAPDH was used as a loading control. (TIF) [file pone.0054342.s004.tif]

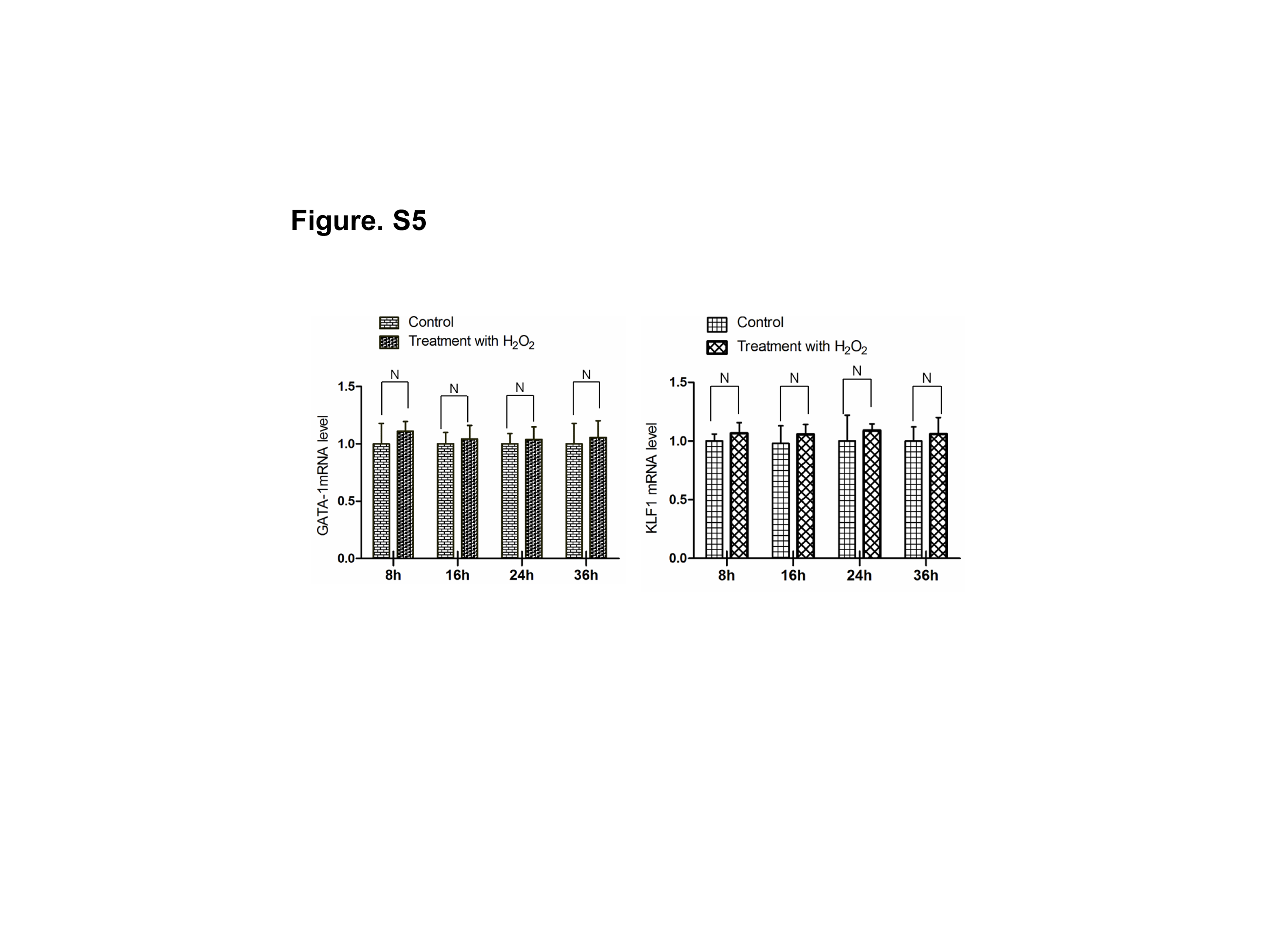

Supplement: Figure S5 — Oxidative stress has no significant effect on the expression levels of GATA-1 and KLF1. Relative GATA-1 and KLF1 mRNA levels determined by qRT-PCR were similar in untreated controls and in SiHa cells treated with H2O2 (1 mM) for 8, 16, 24, or 36 h. Data represent mean ± SD of three RT-PCR reactions (N P>0.05). (TIF) [file pone.0054342.s005.tif]

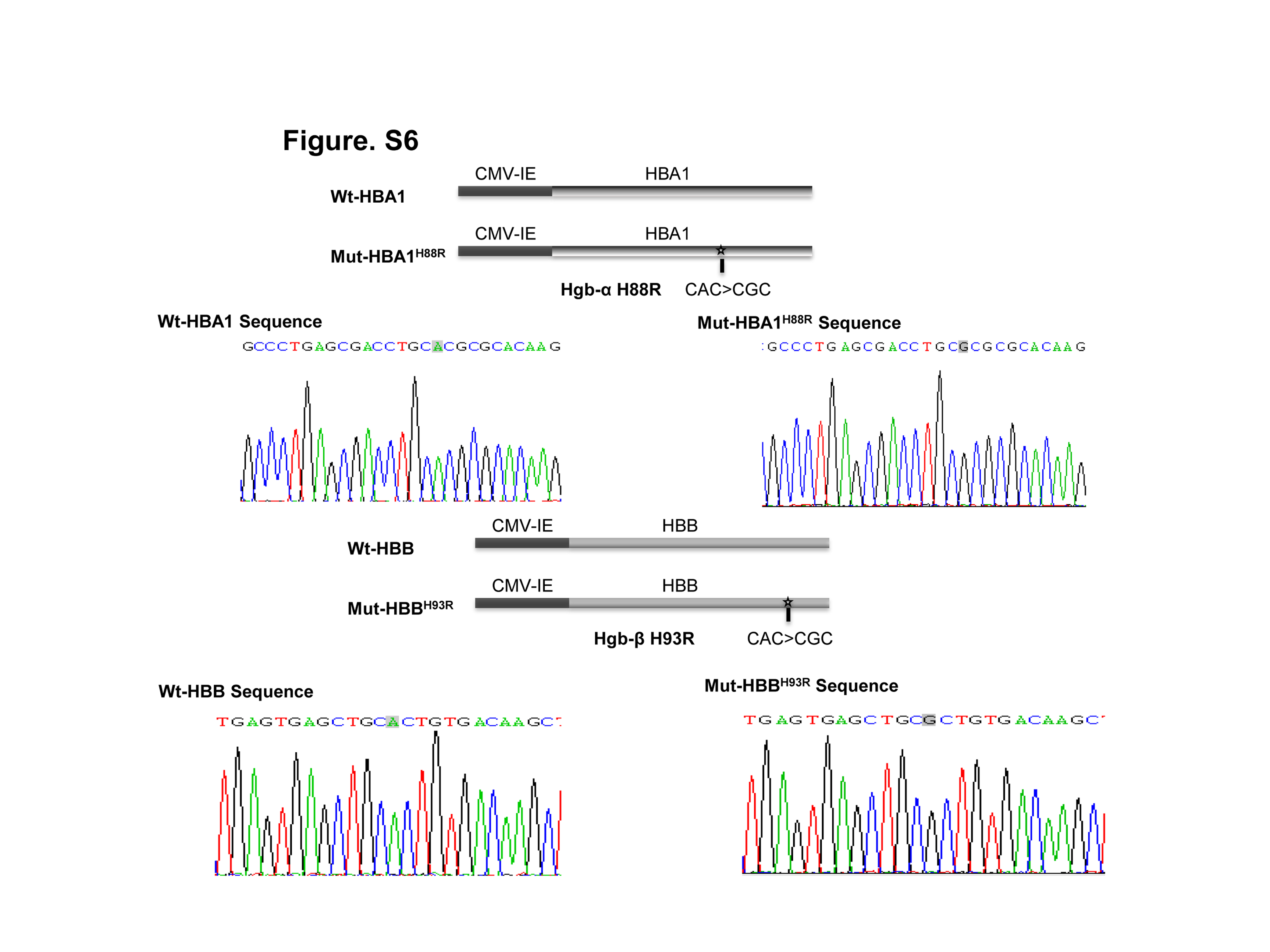

Supplement: Figure S6 — Wild type and mutated Hgb plasmids were verified by sequencing. Schematic representation of the constructions used for overexpressing wild type (HBA1 and HBB) and mutated (HBA1H88R and HBBH93R) Hgb forms in SiHa cells. Black lines represent the CMV-IE promoter, light gray lines represent the HBA1 and HBB genes. All mutation incorporation into the plasmid DNA were verified by sequencing. (TIF) [file pone.0054342.s006.tif]

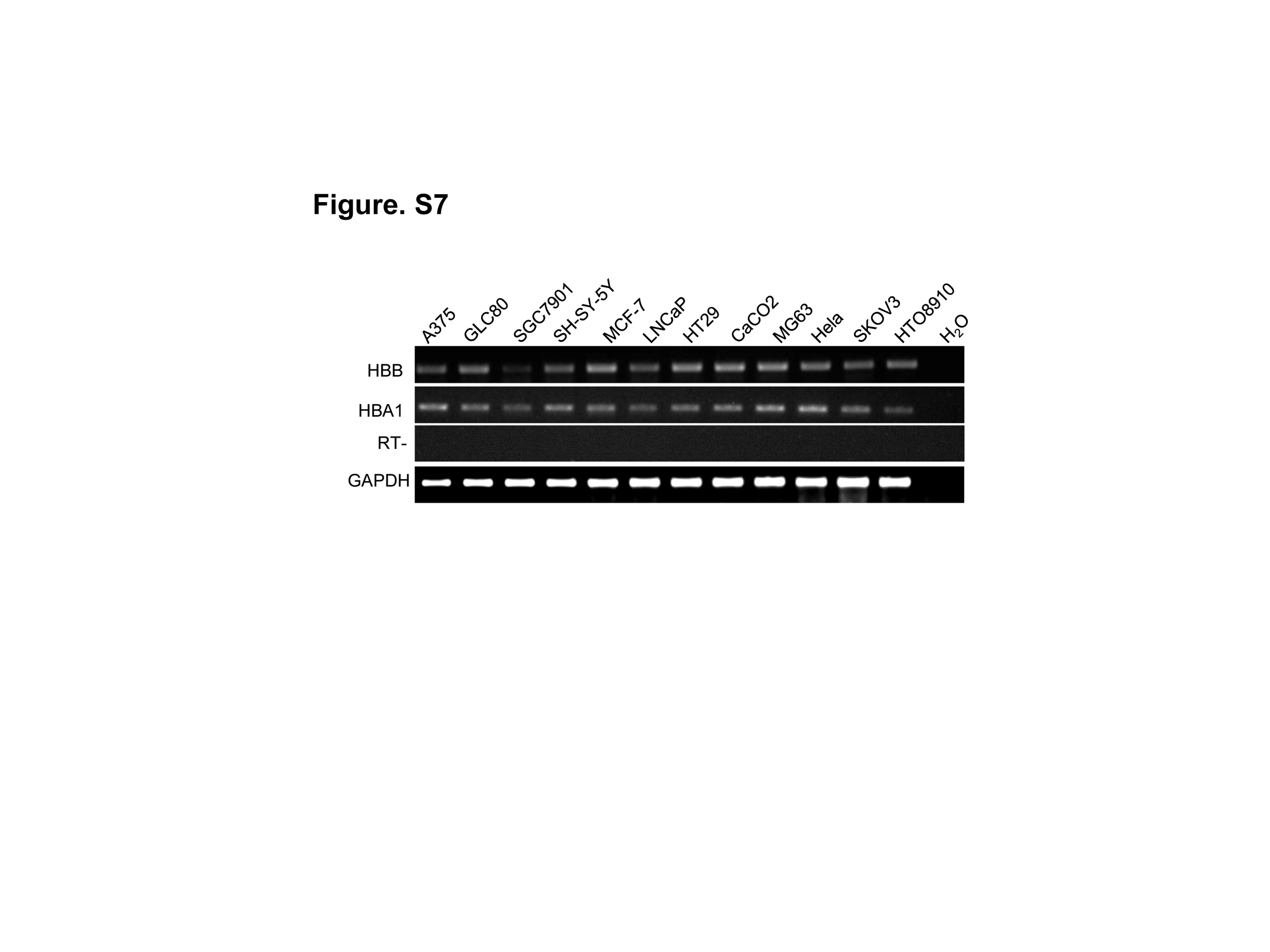

Supplement: Figure S7 — Expression of HBA1 and HBB chains in different solid tumor cell lines. The expression of HBA1 and HBB mRNA was analyzed by RT-PCR. PCR products were separated on 2% agarose gels and visualized with ethidium bromide. Retrotranscriptase free (RT–) as a negative control, H2O, as a system control. (TIF) [file pone.0054342.s007.tif]
